# Editorial: Community series: Spanish Psycholinguistics

**DOI:** 10.3389/fpsyg.2024.1375105

**Published:** 2024-02-19

**Authors:** Jon Andoni Duñabeitia, Pilar Ferré, Isabel Fraga, Mikel Santesteban

**Affiliations:** ^1^Centro de Investigación Nebrija en Cognición, Facultad de Lenguas y Educación, Nebrija University, Madrid, Spain; ^2^Departamento de Psicología, Rovira I Virgili University, Tarragona, Spain; ^3^Cognitive Processes and Behavior Research Group, Department of Social Psychology, Basic Psychology, and Methodology, University of Santiago de Compostela, Santiago de Compostela, Spain; ^4^Department of Linguistics and Basque Studies, University of the Basque Country (UPV/EHU), Vitoria-Gasteiz, Spain

**Keywords:** Spanish Psycholinguistics, Spanish psychology, psycholinguistics, cognition and language, psychology of language

In the domains of linguistic and cognitive research, Spanish Psycholinguistics has emerged as a pivotal area of study, reflecting not only the global prominence of the Spanish language in the scientific arena, but also the increasing academic influence of other regional languages like Catalan, Galician or Basque. The onset of the 21st century marked a significant transition toward the professionalization of experimental research in Spanish Psycholinguistics. This shift has been characterized by the increasing focus of numerous research groups across various nations, delving into the cognitive processes underlying the acquisition, learning, perception, and production of the Spanish language, either as a native or nonnative tongue, as well as of the rest of regional languages used in Spain. Such an expansion of interest and investigation has considerably enriched our understanding of these complex cognitive processes.

The rise of specialized centers and laboratories dedicated to the psycholinguistic and neurolinguistic study of Spanish as a first language, as well as a second, additional, heritage, or foreign language, is a testament to the growing academic and practical interest in this field. Moreover, the recent decades have seen the emergence and consolidation of research groups and centers in Spain, contributing significantly to the country's standing as a key player in the international psycholinguistic arena, as already demonstrated in preceding article collections (see Duñabeitia et al., 2022).

The group of articles included in this Research Topic entitled “*Community Series: Spanish Psycholinguistics*” is a curated collection that aims to enhance the visibility of psycholinguistic research conducted by groups, laboratories, and centers primarily based in Spain or focusing on Spanish or other regional languages spoken in Spain. By including submissions from researchers studying Spanish as a native or non-native language and from groups exploring psycholinguistics in Spain irrespective of their target research language, this volume offers a comprehensive view of the current state and advancements in the field. Besides, this volume plays a crucial role in counteracting the predominantly anglocentric trend in psycholinguistic research. Historically, the field has been heavily centered on English, potentially overlooking the rich linguistic diversity and unique cognitive phenomena present in other languages.

In the article by Casado et al., the authors delve into the intricate relationship between grammatical gender, stereotypes, and gender identity in language processing. Specifically, they studied the processing of gendered words using a gender-priming paradigm. Their results illuminated the multifaceted interplay of linguistic, cognitive, and social factors, significantly contributing to our understanding of gender representations in language.

In the study by Fernández-López et al., the authors challenged conventional notions in the field of visual word recognition by exploring the impact of novel ligatures on word processing. The study employed the masked priming paradigm combined with a lexical decision task, and their results broadened our comprehension of the brain's mechanisms for encoding letters and words, showing that novel ligatures did not significantly distort word processing.

The article by Duñabeitia et al. offers a critical examination of the cognitive processing involved in recognizing words adorned with non-standard diacritics. The study involved a letter detection experiment with both word and nonword stimuli that could include extra non-existent diacritics. This research provides essential insights into the resilience of letter detectors and top-down lexical processes, with far-reaching implications for our understanding of visual word recognition and digital text processing.

The research presented by Andras et al. in their study sheds light on the cognitive benefits of bilingualism. By comparing Spanish–English bilinguals with Spanish monolinguals in a semantic judgment task focusing on the processing of Spanish homophones to produce within-language conflict, they uncover valuable findings regarding the bilingual brain's capacity for conflict resolution and inhibitory control, contributing to the debate on the cognitive impacts of bilingualism.

The article by Demestre explored the use of verb-control information in assigning antecedents to null subjects in sentence processing. The study employed a self-paced reading task in Spanish, manipulating verb type (subject vs. object control) and grammaticality to investigate how quickly verb-specific control information influences the assignment of antecedents. The results suggest that verb control information is rapidly used in the first stages of sentence processing, contributing to our understanding of the cognitive mechanisms involved in language comprehension.

Lastly, the study by Betancourt et al. provides a nuanced understanding of the representation of affective words. The study used a multi-componential approach to identify the characteristics that differentiate emotion-label words (words that explicitly indicate affective states, e.g., *joy*) from emotion-laden words (words that may elicit an emotion, but do not express an emotion directly, e.g., *birthday*). A semantic space was elaborated and a Random Forest Classifier was employed. The results showed that valence is a crucial factor in the organization of the mental lexicon, although other variables related to the multi-componential experience of emotion, like subjective feeling and interoception (internal bodily changes), have a key role in the distinction between emotion-label words and emotion-laden words.

Each article in this Research Topic represents a unique and significant contribution to the field of Spanish Psycholinguistics. This collection of articles not only showcases the vibrant and diverse research within the Spanish psycholinguistics community, but also serves as a foundation for future explorations in the field. It highlights the global significance of the Spanish language in psycholinguistic studies and underscores Spain's role as a leading contributor to this area of research. The collection not only broadens the scope of psycholinguistic research but also offers invaluable insights into the cognitive processing of a language spoken by millions worldwide. This shift is instrumental in fostering a more inclusive conception of psycholinguistics, contributing significantly to the global academic dialogue and enriching our collective knowledge of language processing across diverse linguistic contexts.

## Author contributions

JD: Writing—original draft, Writing—review & editing. PF: Writing—review & editing. IF: Writing—review & editing. MS: Writing—review & editing.
